# A hypernetwork-based urn model for explaining collective dynamics

**DOI:** 10.1371/journal.pone.0291778

**Published:** 2023-09-19

**Authors:** Jiali Lu, Haifeng Du, Xiaochen He

**Affiliations:** 1 School of Public Policy and Administration, Xi’an Jiaotong University, Xi’an, Shaanxi Province, China; 2 School of Economics and Finance, Xi’an Jiaotong University, Xi’an, Shaanxi Province, China; University of Glasgow, UNITED KINGDOM

## Abstract

The topological characterization of complex systems has significantly contributed to our understanding of the principles of collective dynamics. However, the representation of general complex networks is not enough for explaining certain problems, such as collective actions. Considering the effectiveness of hypernetworks on modeling real-world complex networks, in this paper, we proposed a hypernetwork-based Pólya urn model that considers the effect of group identity. The mathematical deduction and simulation experiments show that social influence provides a strong imitation environment for individuals, which can prevent the dynamics from being self-correcting. Additionally, the unpredictability of the social system increases with growing social influence, and the effect of group identity can moderate market inequality caused by individual preference and social influence. The present work provides a modeling basis for a better understanding of the logic of collective dynamics.

## Introduction

Human sociality enables people to perceive and imitate the actions of others, which indicates that individuals are constantly exposed to social influence [1]. Social influence is widely discussed in management, economics, sociology, and psychology, and it is significant in predicting the evolutionary process of political agenda, social networks, and decision-making [2–7]. As a companion of interpersonal interaction, social influence processes such as infection, aggregation, and regulation are bound to occur in individual behaviors. Collective dynamics refer to those behaviors that occur in response to a common social influence or stimulus under relatively spontaneous, unpredictable, and unstable circumstances, which is a class of high-risk and extremely destructive social events [8]. The collective dynamics of social influence are obtaining significant attention and have significant research implications in the realm of sociology [9, 10], psychology [11, 12], and anthropology [13–15]. For example, individuals may consider the choices of early decision-makers in the market, i.e., accessing the products’ popularity information as the criterion for their choices [16]. As a result, individuals may choose the same decision as others, which creates a self-reinforcing path of collective dynamics [17, 18].

Computational social science opens a door to better describing and predicting human social interactions, as well as providing modeling approaches for complex and intractable social issues. Aiming to explore the detailed characteristics of collective dynamics, a range of computational and statistical models have been explored [19, 20]. A popular scheme that is widely discussed in the literatures to explain the social influence process is the urn model [21, 22], which is proposed by Pólya [23]. The classic Pólya urn model is a simple stochastic process based on a ball-drawing process, at each time, a ball is randomly drawn from the urn and then return along with a ball of the same color. Since balls with the majority color are more likely to be drawn, resulting in the majority color gradually dominating the entire urn, which forms a self-reinforcement path for the dynamics of social influence [18, 24–26]. Urn model has been extensively applied to delineate diverse phenomena [27], including the species evolution process [28], game strategy and decision-making [29], the dynamics of novelties and innovations [30], tourism activities and voting forecasting [31, 32].

In addition to the dynamic process of social systems, the depiction of static social structures also receives a surge of attention from the academic community. Complex networks like scale-free networks [33], correlation networks [34], dynamic networks [35], and other models of information dissemination have contributed to the interpretation of collective dynamics. However, the use of simple or directed graphs to illustrate complex networks cannot provide a complete description of real-world systems, especially in the study of connectivity, clustering, and other topological properties [36]. Besides, not all human interactions happen directly point-to-point, instead, there are many other modes of social influence such as domain-aware influential power [37], combinatorial theories of consensus production [38], collective cooperation and battle [39]. In particular, the prevailing of social media greatly facilitates people to exchange and spread information and favors the formation of like-minded users’ groups or communities framing then reinforcing shared perspectives [40]. The way people receive information is no longer individual to individual, a case in point is when someone posts a moment in WeChat, all of his friends are able to receive that information. Another instance is that collaborations in the scientific community are in fact not simply collaborations between two researchers, but between several research teams. Therefore, complex networks based on simple graphs are no longer applicable for performing and computing collective dynamics in complex social systems. Social relations with group bonds require new forms of network structure depictions to express their properties and behaviors [41].

A reasonable solution is to construct "networks of networks" using the relevant theory of hypergraphs [42]. Berge first proposed the concept of hypergraphs [43]. Ghoshal et al. proposed a mathematical model for random hypergraphs [44], and the topological characteristics of hypergraphs have subsequently been widely discussed [45, 46]. Estrada and Rodríguez-Velázquez extended the concept that hypergraph clustering for complex networks can be defined as hypernetworks [36]. The significant contribution of hypernetworks is the construction of a third dimension of user labeling based on the binary structure of individuals and pairwise relationships. Bodó et al. extended the epidemic propagation model to hypernetworks and enabled higher accuracy of the epidemic model [47]. Other studies like collective cooperation [48], ecological networks [49], and disease predictions [50] are proven high accuracy utilizing hypernetwork structures. Hypernetworks have currently been broadly applied to model social media networks [41], music recommendation systems [51], choice dilemmas [52], and other dynamic models [53, 54].

Unlike the physical world, social systems are subject to dynamic changes, which require constantly updated theories to support public policy and administration [55]. Modeling of collective dynamics provides a better understanding of the fundamental principles of collective dynamics, and yields governments with crucial policy recommendations to properly avoid highly risky issues. In what follows, the present study introduces a hypernetwork-based urn model with theoretical derivation of collective dynamics in Section II. Section III displays our simulations and experiments. Finally, Section IV presents our conclusions and discussions.

## Methodology

Salganik et al. proposed a “independent world versus influenced world” framework based on an artificial music market experiment [16]. In an independent world, people make their decisions merely based on personal preferences. However, in a thoroughly influenced world, people will follow others’ trails. Due to social influence, human interactions become intertwined and individuals are no longer independent of each other. Social influence has long been extensively studied because of its potential to aggregate collective behaviors, which may have dramatic consequences in economics, politics and cultures. Specifically, social influence in market system can form a path-dependent process in which the early popularity advantage of an object can be perpetuated by driving feedback, thus affecting later individuals’ choices [26].

**Fig 1:**
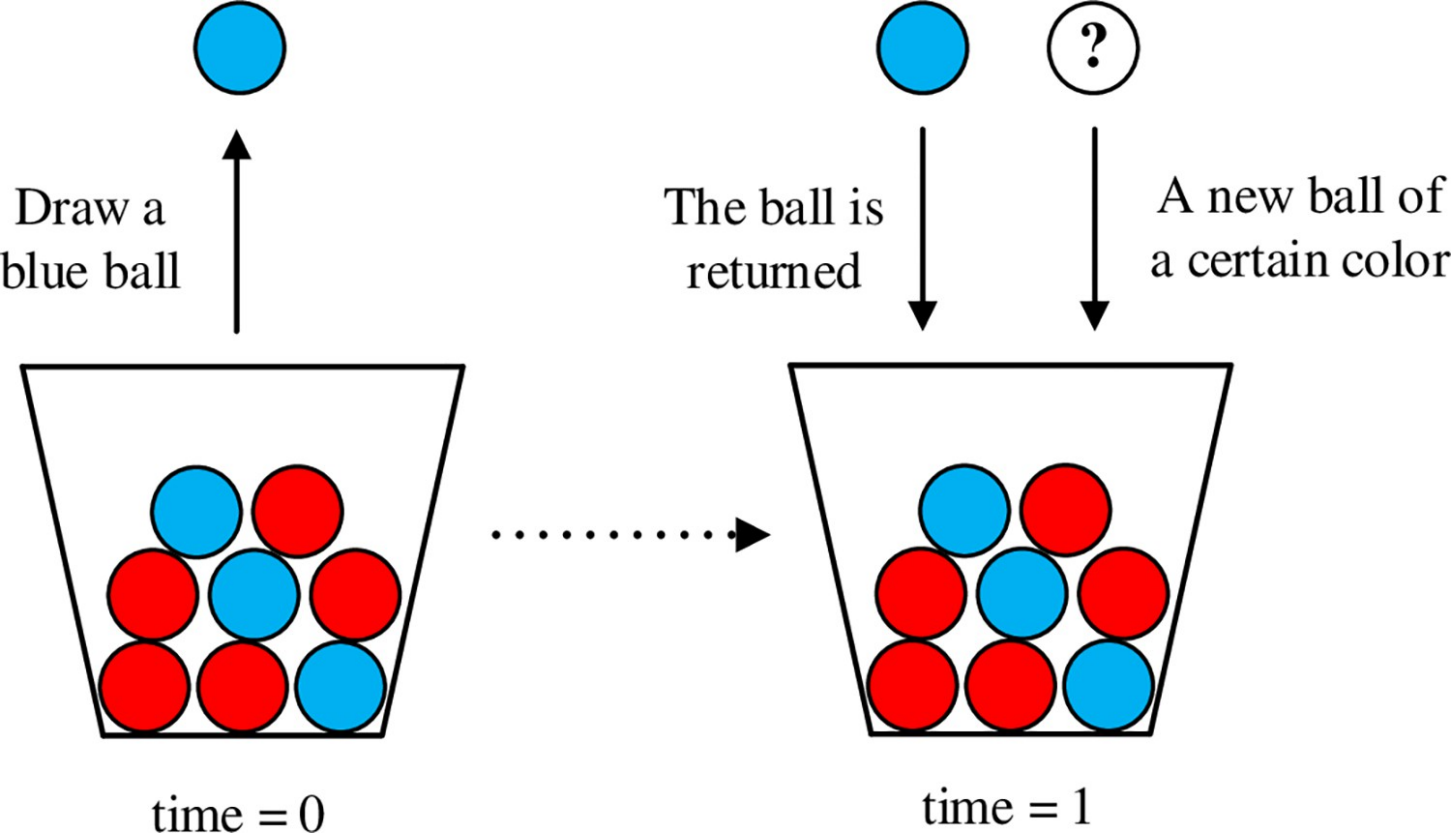
An illustration of the urn model. Individuals will make choices (choose red or blue balls) by observing others’ actions.

The traditional Pólya urn model generally demonstrates the *rich get richer* phenomenon, which precisely demonstrates a path-dependent process [27]. In the initial state, *Ir*_(0)_ red balls and *I*_*b*(0)_ blue balls are in the urn. At each step, a ball is inspected randomly from the urn and a ball with the same color is added into it [56]. Since the Pólya urn offers a simple framework for modeling, some variants of the urn model have been subsequently proposed [57–59], e.g., more balls are drawn or added at each step, more colors of balls are included, and more complex dynamic strategies are applied. In our model, we follow the basic ideas of the Pólya urn model. The self-reinforcement process of the Pólya urn model is consistent with the evolution of collective dynamics. We suppose there are two types of balls in the market, randomly draw a ball then put a new ball of the same or opposite color according to certain rules. An illustration of our model is shown in Fig 1.

With the rise of network analysis, there has been growing interests in studying social structures instead of simply macro-perspective. In contrast with mean-field approaches, such as the network structure of the classical urn model, where all individuals are essentially connected with each other, network-based approaches assume that individuals are the nodes of a network and are connected by direct, binary connections through edges [60]. Therefore, complex networks can provide a more accurate and vivid depiction of human relationships with respect to the fully-connected network.

While in the real-world system, most of the actions taken by individuals are commonly influenced by their organizations or groups [61]. We can resort to the group identity theory to interpret this phenomenon [62]. Group identity refers to an individual’s awareness of belonging to a certain group and having a psychological attachment to that group based on a perception of shared beliefs, feelings, interests, and ideas with other group members [63–65]. Individuals will derive self-esteem from that group membership and behave uniformly according to the group norms [66]. Hence, organizations or groups structured by relational ties is a necessary carrier of contagion in the process of social influence [67].

**Fig 2:**
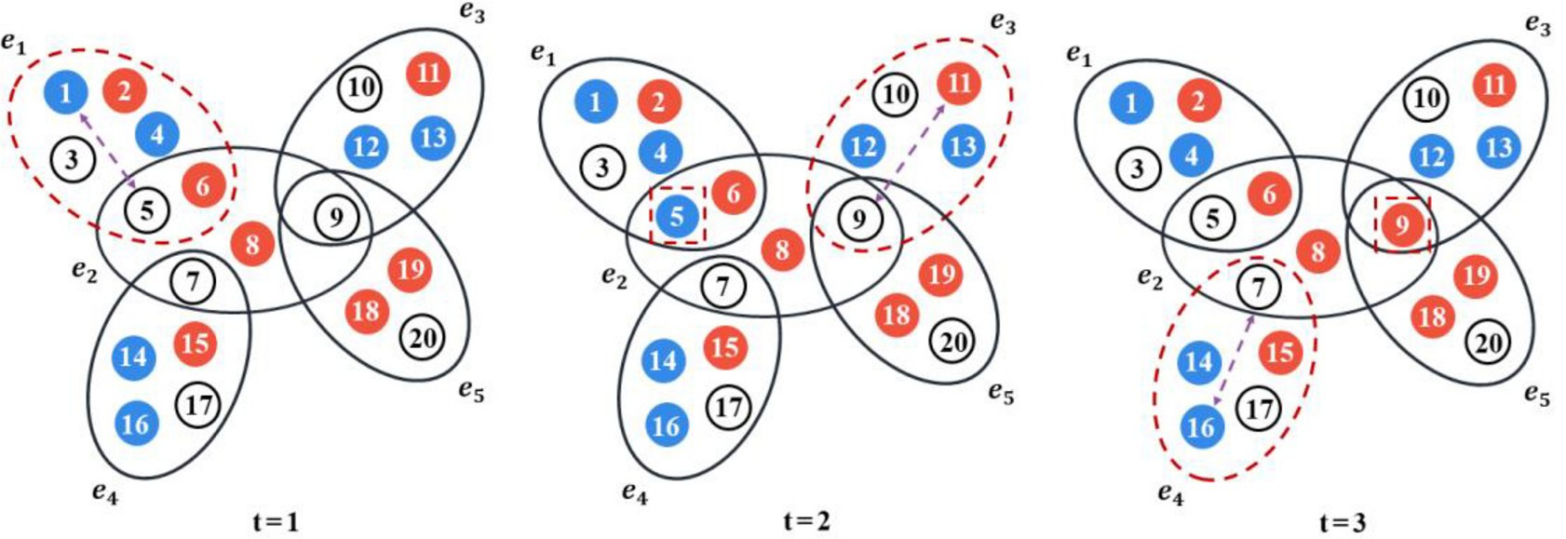
Schematic illustration of the dynamic process on our hypernetwork-based urn model. When time = 2, individual 5 has already made a choice at time 1, and individual 9 will make a choice based on the state of individual 11.

With the consideration of the group identity effect, we propose a new form of the urn model by extending the Pólya urn process to hypernetworks. In a graph, an edge is associated with two nodes, but a hyperedge in a hypergraph can connect an arbitrary number of nodes. Hypernetwork is a remarkably common relationship pattern in social systems, it consists of individuals linked by common membership in organizations, or of organizations linked by individuals who belong to several organizations at once [68]. In our hypernetwork, a node represents an individual, and a hyperedge represents a group such as workplace, community, or political parties. In conjunction with the urn model, individuals will be influenced by individuals from the same group or the group itself. Compared with the classic urn model, hypernetwork-based urn model may generate a completely different mechanism because groups can scale their value to the entire population through hyperedges [69]. Our hypernetwork structure can be expressed as follows, let *X = {x*_1_, *x*_2_, *…*, *x*_*n*_*}* be a finite individual set, the group structures on individuals *X* is a family *E = {e*_1_, *e*_2_, *…*, *e*_*m*_*}* of subsets of *X*. Each hyperedge has a potential norm, denoted as *G = {g*_1_, *g*_2_, *…*, *g*_*m*_*}*. Assume that the dynamic evolution occurs on a uniform hypernetwork, and the illustration of our model is shown in Fig 2.

**Fig 3:**
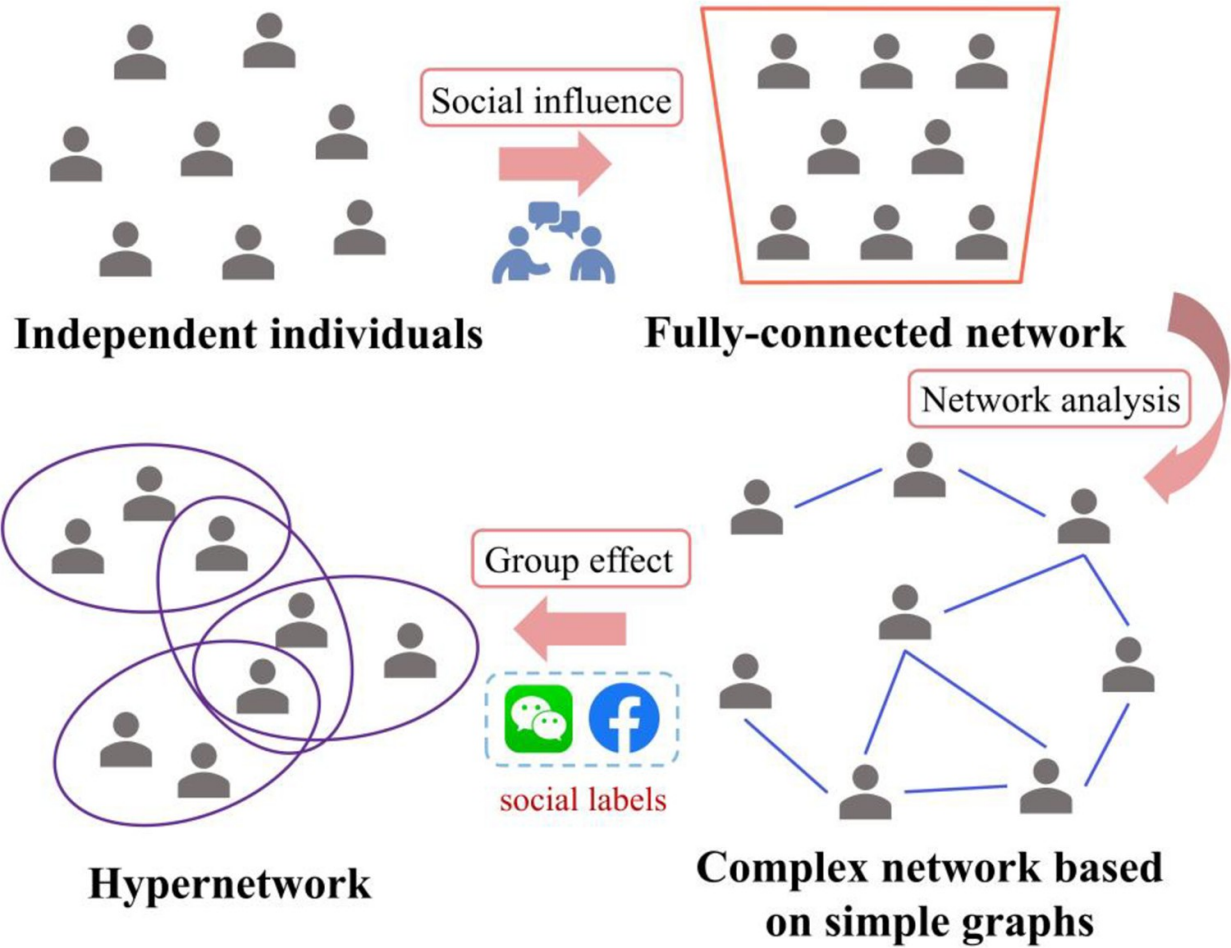
Schematic illustration of social network structure of human interaction. Social influence allows individuals to perceive external information instead of being independent, then network analysis offers a heightened precision in delineating the intricate configurations underlying human interaction patterns. Furthermore, group effects elucidate various groups in social interaction, subsequently forming hypernetworks.

The network structure transformation of human interaction mentioned above can be depicted in Fig 3. Social influence allows individuals to perceive external information, as in the classical urn model, where individuals are no longer isolated from each other. The prevalence of network analysis leads to a more realistic and detailed description of human interaction structures in social systems, prominently represented by complex networks based on pairwise relationships. However, the flourishing of social media has greatly facilitated interpersonal communication and various groups have emerged. The hypernetwork structure can better demonstrate this third dimension of labeling in addition to individuals and relationships. A fully-connected network is embedded in each hyperedge, and individuals located in the same group have access to information from group members. In this paper, the presented urn model in hypernetwork structure is capable of exploring group identity effect, where the nodes signify individuals and the hyperedges represent the organizations or groups.

We refer to the extent of dependence among individuals as the social influence *p*_*s*_, in other words, the probability of an individual imitating others is *p*_*s*_. Next, to explore the effect of group identity on individuals’ decisions, we assume that each hyperedge has an initial purpose. Namely, each hyperedge has a corresponding specification, and individuals in this hyperedge are required to comply with this norm. We define the probability that the initial purpose of a hyperedge is red balls as *δ*_*1*_, and the probability that an individual’s decision is consistent with the initial purpose of a particular hyperedge is *p*_*c*_. In the dynamic process of decision-making, people do not solely follow the crowd or groups, but may also consider their own preferences. Thus, we set individual’s personal preference as *q*_*s*_.

The procedure of our proposed hypernetwork-based urn model is constructed in our algorithm. We set a global urn for the whole system and a local urn which is represented as hyperedges for each individual *l*. In the dynamic process, people can only acquire messages from their local urns. For each *l*’s neighbor *k*, *k* puts its color choice into *l*’s local urn, and if *k* has not made a decision, it will not place any balls in *l*’s urn. Next, individual *l* selects a ball from its local urn and with a probability of social influence *p*_*s*_ places a ball of the same color into the global urn. While with a probability of 1 *− p*_*s*_, individual *l* first makes a choice according to the initial purpose of the hyperedge with a probability of *p*_*c*_, otherwise it will select a color ball depending on their personal preference *q*_*s*_. If individual *l*’s local urn is an empty urn, namely none of *l*’s neighbors have already made decisions, then we should randomly select another individual *l* and duplicate the above dynamic process.

**Algorithm Ⅰ.** The procedure of the hypernetwork-based urn model with group identity effect

1. input: *m* (number of individuals), *n* (number of hyperedges), δ_1_(probability of hyperedge possessing a red purpose), *p*_*s*_ (probability of social influence), *p*_*c*_ (probability of complying with group norms), *q*_*s*_ (personal preference for choosing red balls);

2. Randomly generate a uniform hypernetwork with *m* nodes and *n* hyperedges, initialize the hyperedges with different initial purposes (color *i* or color *j*) according to δ_1_, and each purpose’s count $E_{\left(0\right)}=[E_{i(0)},E_{j(0)}]$; initialize a global *U*rn with existing choices and each color’s count $N_{\left(0\right)}=[I_{i(0)},I_{j(0)}]$;

3. while all individuals have made decisions:

4.  At time *s*, randomly select an individual *l* who hasn’t made choice, then select an individual *k* located in the same hyperedge as *l* and has already made a decision (if there is no one who has made decision in *l*’s local urn, randomly select another individual *l*);

5.  if *Urn*_(*l*)_ is not an empty urn

6.    if randomly generated *r*_*1*_ < = *p*_*s*_

7.      *N*_*k*(*s*)_ = *N*_*k*(*s*-1)_+1;

8.      *Urn* = [*Urn*, *k*];

9.    else

10.      if randomly generated *r*_*2*_ < *p*_*c*_

11.      pick color *h* based on this hyperedge’s initial purpose;

12.      *N*_*h*(*s*)_ = *N*_*h*(*s*-1)_+1;

13.      *Urn* = [*Urn*, *h*];

14.      else

15.      pick a ball with color *g* that is individual *l*’s preferred color based on *q*_*s*_;

16.      *N*_*g*(*s*)_ = *N*_*g*(*s*-1)_+1;

17.      *Urn* = [*Urn*, *g*];

18.    end

19.  else

20.    randomly select another individual *l* and duplicate the above dynamic process;

21.  end

22. Output: *Urn* and *N*_(*n*)_.

To generally understand the effect of group identity on individuals’ decision-making, we simplify the relationship of nodes and performed a theoretical derivation. We focus simply on the collective dynamics in one certain hyperedge and extrapolate the evolution of the whole hypernetwork based on that. The probability of the hyperedge’s purpose initialized as color *i* is *δ*_*i*_, and the probability that individuals comply with this group norm is *p*_*c*_. Then, we can compute the intrinsic value preference *q*_*i*_ for color *i* (the market share of color *i* in the independent world). Assuming the initial urn is an empty urn, the probability of choosing a ball with color *i* by the first individual is $PS_{i(1)}^{1}=\delta_{i}p_{c}+(1-p_{c})q_{i}$, while the probability of choosing another color is $PS_{i(1)}^{0}=(1-\delta_{i})p_{c}+(1-p_{c})(1-q_{i})$. Assuming that the probability of the social influence is *p*_*s*_, when a new individual enters the system, the probability of not choosing color *i* will be $PS_{i}^{0}{}_{\left(2\right)}=PS_{i}^{0}{}_{\left(1\right)}[p_{s}+(1-p_{s})(\left(1-\delta_{i})p_{c}+(1-p_{c})(1-q_{i}\right))]$, the probability of choosing color *i* once will be $PS_{i}^{1}{}_{\left(2\right)}=(1-p_{s})[PS_{i}^{1}{}_{\left(1\right)}\cdot (\left(1-\delta_{i})p_{c}+(1-p_{c})(1-q_{i}\right))+PS_{i}^{0}{}_{\left(1\right)}(\delta_{i}p_{c}+(1-p_{c})q_{i})]$, and the probability of choosing *i* twice will be $PS_{i}^{2}{}_{\left(2\right)}=PS_{i}^{1}{}_{\left(1\right)}[p_{s}+(1-p_{s})(\delta_{i}p_{c}+(1-p_{c})q_{i})]$. When three individuals enter the dynamic, the probability of not choosing color *i* is $PS_{i}^{0}{}_{\left(3\right)}=PS_{i}^{0}{}_{\left(2\right)}[p_{s}+(1-p_{s})(\left(1-\delta_{i})p_{c}+(1-p_{c})(1-q_{i}\right))]$, while choosing *i* once is $PS_{i}^{1}{}_{\left(3\right)}=PS_{i}^{1}{}_{\left(2\right)}[\frac{1}{2}p_{s}+(1-p_{s})(\left(1-\delta_{i})p_{c}+(1-p_{c})(1-q_{i}\right))]+PS_{i}^{0}{}_{\left(2\right)}(1-p_{s})[\delta_{i}p_{c}+(1-p_{c})q_{i}]$, that of choosing color *i* twice is $PS_{i}^{2}{}_{\left(3\right)}=PS_{i}^{2}{}_{\left(2\right)}[\left(1-p_{s})(\left(1-\delta_{i})p_{c}+(1-p_{c})(1-q_{i}\right)\right)]+PS_{i}^{1}{}_{\left(2\right)}[\frac{1}{2}p_{s}+(1-p_{s})(\delta_{i}p_{c}+(1-p_{c})q_{i})]$, and choosing color *i* three times is $PS_{i}^{3}{}_{\left(3\right)}=PS_{i}^{2}{}_{\left(2\right)}\cdot [p_{s}+(1-p_{s})(\delta_{i}p_{c}+(1-p_{c})q_{i})]$. When there are *n* (*n*≥2 individuals, the probability of choosing *i* can be formulated as Eq (1).


$$\begin{array}{l} PS_{i}^{0}{}_{\left(n\right)}=PS_{i}^{0}{}_{\left(n-1\right)}[p_{s}+(1-p_{s})(\left(1-\delta_{i})p_{c}+(1-p_{c})(1-q_{i}\right))]\\ PS_{i}^{1}{}_{\left(n\right)}=PS_{i}^{1}{}_{\left(n-1\right)}[\frac{n-2}{n-1}p_{s}+(1-p_{s})(\left(1-\delta_{i})p_{c}+(1-p_{c})(1-q_{i}\right))]+PS_{i}^{0}{}_{\left(n-1\right)}[\left(1-p_{s})(\delta_{i}p_{c}+(1-p_{c})q_{i}\right)]\\ PS_{i}^{2}{}_{\left(n\right)}=PS_{i}^{2}{}_{\left(n-1\right)}[\frac{n-3}{n-1}p_{s}+(1-p_{s})(\left(1-\delta_{i})p_{c}+(1-p_{c})(1-q_{i}\right))]+PS_{i}^{1}{}_{\left(n-1\right)}[\frac{1}{n-1}p_{s}+(1-p)(\delta_{i}p_{c}+(1-p_{c})q_{i})]\\ \vdots \\ PS_{i}^{n-2}{}_{\left(n\right)}=PS_{i}^{n-2}{}_{\left(n-1\right)}[\frac{1}{n-1}p_{s}+(1-p_{s})(\left(1-\delta_{i})p_{c}+(1-p_{c})(1-q_{i}\right))]+PS_{i}^{n-3}{}_{\left(n-1\right)}[\frac{n-3}{n-1}p_{s}+(1-p_{s})(\delta_{i}p_{c}+(1-p_{c})q_{i})]\\ PS_{i}^{n-1}{}_{\left(n\right)}=PS_{i}^{n-1}{}_{\left(n-1\right)}[\left(1-p_{s})(\left(1-\delta_{i})p_{c}+(1-p_{c})(1-q_{i}\right)\right)]+PS_{i}^{n-2}{}_{\left(n-1\right)}[\frac{n-2}{n-1}p_{s}+(1-p_{s})(\delta_{i}p_{c}+(1-p_{c})q_{i})]\\ PS_{i}^{n}{}_{\left(n\right)}=PS_{i}^{n-1}{}_{\left(n-1\right)}[p_{s}+(1-p_{s})(\delta_{i}p_{c}+(1-p_{c})q_{i})] \end{array}$$ (1)

We can simplify Eq (1) can as Eq (2), where $PS_{i}^{j}{}_{\left(n\right)}$ denotes the probability of choosing color *i j* times when there are *n* individuals:

$$\begin{array}{l} PS_{i}^{j}{}_{\left(n\right)}=PS_{i}^{j}{}_{\left(n-1\right)}[p_{s}+(1-p_{s})(\left(1-\delta_{i})p_{c}+(1-p_{c})(1-q_{i}\right))]\;if\;j=0\\ PS_{i}^{j}{}_{\left(n\right)}=PS_{i}^{j}{}_{\left(n-1\right)}[\frac{n-1-j}{n-1}p_{s}+(1-p_{s})(\left(1-\delta_{i})p_{c}+(1-p_{c})(1-q_{i}\right))]\\ \;+PS_{i}^{j-1}{}_{\left(n-1\right)}[\frac{j-1}{n-1}p_{s}+(1-p_{s})(\delta_{i}p_{c}+(1-p_{c})q_{i})]\;if\;1\leq j\leq n-1\\ PS_{i}^{j}{}_{\left(n\right)}=PS_{i}^{j-1}{}_{\left(n-1\right)}[p_{s}+(1-p_{s})(\delta_{i}p_{c}+(1-p_{c})q_{i})]\;if\;j=n \end{array}$$ (2)

Then, supposed that the initial urn is not an empty urn, the network originally has *B* individuals and *B*_*i*_ individuals have already chosen color *i* as their decision. The probability of *j*+*B*_*i*_ people choosing color *i* after *n* new individuals entering the market can be formulated as Eq (3).


$$\begin{array}{l} PS_{i}^{j+B_{i}}{}_{\left(n+B\right)}=PS_{i}^{j+B_{i}}{}_{\left(n-1+B\right)}[\frac{n-1+B-B_{i}}{n-1+B}p_{s}+(1-p)(\left(1-\delta_{i})p_{c}+(1-p_{c})(1-q_{i}\right))]\;if\;j=0\\ PS_{i}^{j+B_{i}}{}_{\left(n+B\right)}=PS_{i}^{j+B_{i}}{}_{\left(n-1+B\right)}[\frac{n-1+B-j-B_{i}}{n-1+B}p_{s}+(1-p_{s})(\left(1-\delta_{i})p_{c}+(1-p_{c})(1-q_{i}\right))]\\ \;+PS_{i}^{j-1+B_{i}}{}_{\left(n-1+B\right)}[\frac{j-1+B_{i}}{n-1+B}p_{s}+(1-p_{s})(\delta_{i}p_{c}+(1-p_{c})q_{i})]\;if\;1\leq j\leq n-1\\ PS_{i}^{j+B_{i}}{}_{\left(n+B\right)}=PS_{i}^{j-1+B_{i}}{}_{\left(n-1+B\right)}[\frac{j-1+B_{i}}{n-1+B}p_{s}+(1-p_{s})(\delta_{i}p_{c}+(1-p_{c})q_{i})]\;if\;j=n \end{array}$$ (3)

We can use Eq (3) to obtain each color *i*’s market share *M*_*i*_ = *N*_*i*_/*n*, where *N*_*i*_ is the number of individuals selecting color *i* in the network. When there are *n*+*B* individuals enter the dynamic, the expected value for color *i*’s market share should be formulated as $E\left[M_{i}{}_{\left(n+B\right)}\right]=\left[\sum_{j=0}^{n}\left[PS_{i}^{j+B_{i}}{}_{\left(n+B\right)}\cdot (j+B_{i})\right]\right]/\left(n+B\right)$. To calculate the stability and convergence of the dynamic, we made a simple transformation to the above mathematical expectation formula. Under the condition of the known market share of color *i* at time *t* = *n*−1, the probability of the next individual choosing color *i* is $E[P_{i(n)}]=p_{s}M_{i(n-1)}+(1-p_{s})(\delta_{i}p_{c}+(1-p_{c})q_{i})$. Correspondingly, the expected value of color *i*’s market share is given in Eq (4).


$$E[M_{i(n+B)}]=\frac{N_{i(n-1+B)}+1}{n+B}P_{i(n)}+\frac{N_{i(n-1+B)}}{n+B}(1-P_{i(n)})$$ (4)

According to above derivations, we substitute *P*_*i*(*n*)_ into Eq (4), therefore the expected value of the market share of color *i* can be transformed as recursive Eq (5).


$$E[M_{i(n+B)}]=(1-\frac{1-p_{s}}{n+B})M_{i(n-1+B)}+(1-p_{s})\frac{\delta_{i}p_{c}+(1-p_{c})q_{i}}{n+B}$$ (5)

Depending on Eq (5), when *n* is infinite, it can be considered that $\underset{n\to \infty }{\lim}M_{i(n+B)}=M_{i(n-1+B)}$ and we can acquire $\frac{1-p_{s}}{n+B}M_{i(n+B)}=(1-p_{s})\frac{\delta_{i}p_{c}+(1-p_{c})q_{i}}{n+B}$. Apparently, when *p*_*s*_ = 1, *M*_*i*(*n*+*B*)_ will converge to an arbitrary value, which is consistent with the conclusion of the classic urn model [23]. While *p*_*s*_<1, *M*_*i*(*n*+*B*)_ will converge to $\delta_{i}p_{c}+(1-p_{c})q_{i}$. Obviously, the market share of color *i* increases with *δ*_*i*_, and as *p*_*c*_ gets larger, the proportion of color *i* grows more rapidly, which is consistent with group identity theory [70]. This convergence value can be rewritten as $M_{i(n+B)}=(\delta_{i}-q_{i})p_{c}+q_{i}$, which is a linear function for *p*_*c*_ when the value of *δ*_*i*_ is determined. If *δ*_*i*_<*q*_*i*_, *M*_*i*(*n*+*B*)_ is a linear function with a negative slope; if *δ*_*i*_ = *q*_*i*_, the market share of color *i* will converge to be consistent with the individual preference value *q*_*i*_; while if *δ*_*i*_>*q*_*i*_, it linearly increases as *p*_*c*_ increases. The results indicate that the group norms in hypernetwork play an essential role in the evolution of collective dynamics. The effect of group identity can promote self-correcting of radical individual preference.

## Experiments

To further explore the evolution of collective dynamics, we conducted a series of simulation experiments. The experiments are implemented based on our algorithm, and the procedure is written and carried out by MATLAB R2022b. To eliminate the random effects, each simulation is carried out for 500 iterations, and the results are obtained by averaging over the independent iterations. In order to present a dynamic market consisting of products with different innate qualities, we assume that there are two colors of balls (red and blue), and the qualities of the red and blue balls are given as *q*_1_ = 1 and *q*_2_ = 0, respectively. Other necessary parameters used in the experiments are summarized in Table 1. To explore the effect of initial bias, we initially place ten balls in the urn and set three different start conditions: a false start with one red ball and nine blue balls in the urn, an equal start with five red balls and five blue balls in the urn, and a correct start with nine red balls and one blue ball in the urn at the beginning. These ten initial nodes are randomly selected in each run so as to cover different influence paths.

**Table 1:** Parameters used in the simulation experiments.

Parameter	Meaning	Value Range
*p*_*s*_	Probability of social influence	0–1
*p*_*c*_	Probability of conformity to group norms	0–1
*δ*_1_	Probability that the initial purpose of a hyperedge is red	0–1

**Fig 4:**
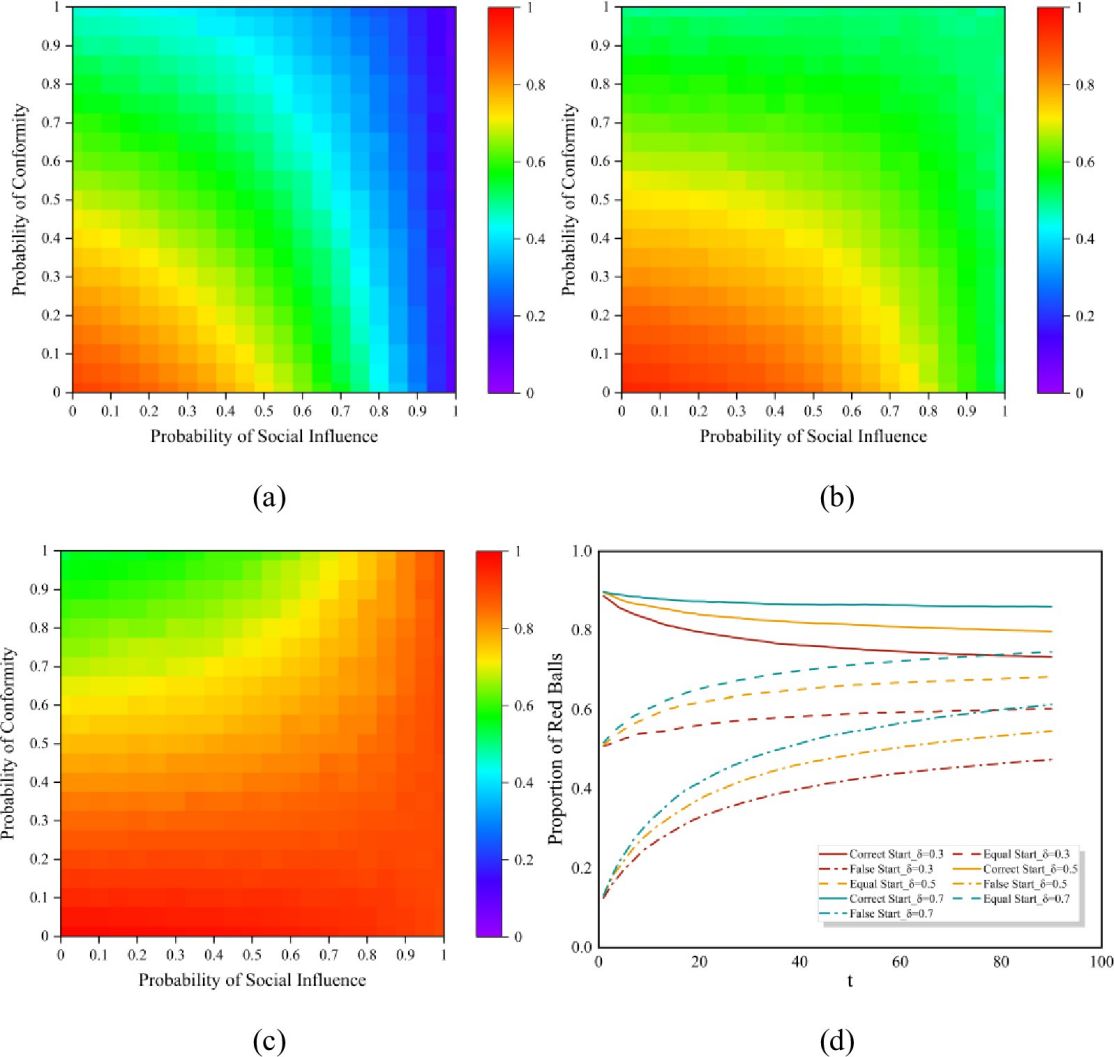
Proportion of the red balls with different probability of social influence *p*_*s*_ and probability of conformity *p*_*c*_. (a) is the result with a false start in a hypernetwork with *δ*_1_ = 0.5; (b) is an equal start in a hypernetwork with *δ*_1_ = 0.5; (c) is a correct start in a hypernetwork with *δ*_1_ = 0.5; (d) is the timeline result with *p*_*s*_ = 0.5, *p*_*c*_ = 0.5.

Since social members have relatively stable relations in common issues, we assume the hypernetwork structure is static [71], and the main structural parameters of our hypernetwork are as follows: there are 100 nodes, and 15 hyperedges in the hypernetwork. Each hyperedge contains 10 nodes, which indicates that this hypernetwork is a uniform hypernetwork. Fig 4 shows the crossover impact of the probability of conformity *p*_*c*_ and the probability of social influence *p*_*s*_ on the dynamics under our algorithm. We first set *δ*_1_ = 0.5, namely, the probability of hyperedges with the initial preference of red or blue ball is identical. The heatmaps display that the proportion of the red ball spreads out in waves, and it is higher when both *p*_*s*_ and *p*_*c*_ are small. When the probability of social influence *p*_*s*_ is small, the red balls will completely dominate the market because of the personal preference. The proportion of the red ball gets smallest when there are one red ball and nine blue balls at the initial state, indicating that the initial state largely determines the results, and a larger social influence can adjust the results to the initial state.

However, regardless of the initial state, the market share of the red balls tends to converge to *δ*_1_ as *p*_*c*_ rises. Smaller *p*_*c*_ indicates a weakened concept of group and the lack of group cohesion. When both *p*_*s*_ and *p*_*c*_ are small, the individuals’ decisions will depend on their own preferences *q*_1_ = 1. Thus, there is a distinct red triangle in the lower left corner of Fig 4A and 4B. When there are nine red balls and one blue ball in the initial state, the results present a green upper left region, confirming that large probability of conformity *p*_*c*_ will regulate the market share to equality when *δ*_1_ = 0.5, as shown in Fig 4C.

In this case, we also observed that when the social influence *p*_*s*_ is very large, the final results will always be close to the initial state no matter how *p*_*c*_ changes. This self-reinforcement process suggests that a larger *p*_*s*_ will weaken the effect of group identity of the hypernetwork, as well provide a strong imitation environment, which may prevent the dynamics from being self-correcting. While higher probability of conformity *p*_*c*_ will lead the results converge to the initial purpose probability *δ*_1_ of the hypernetwork, which still confirms the self-reinforcement process.

Next, we varied the value of *δ*_1_ to investigate how the effect of group identity affect the dynamics. Fig 4D depicts the market share results at each moment. The horizontal coordinate in the graph is the propagation time *t* and we set the completion of one propagation as one time unit. We set *δ*_1_ = 0.3, *δ*_1_ = 0.5 and *δ*_1_ = 0.7 respectively and obtained the results under the three different initial starts. Obviously, when *δ*_1_ is larger, the proportion of red balls will always be higher under any initial state, as indicated by the green line in Fig 4D. It suggests that the sense of group identity will lead individuals toward a consistent goal, which may facilitate market equality when initial state and the group norms of the market differ.

**Fig 5:**
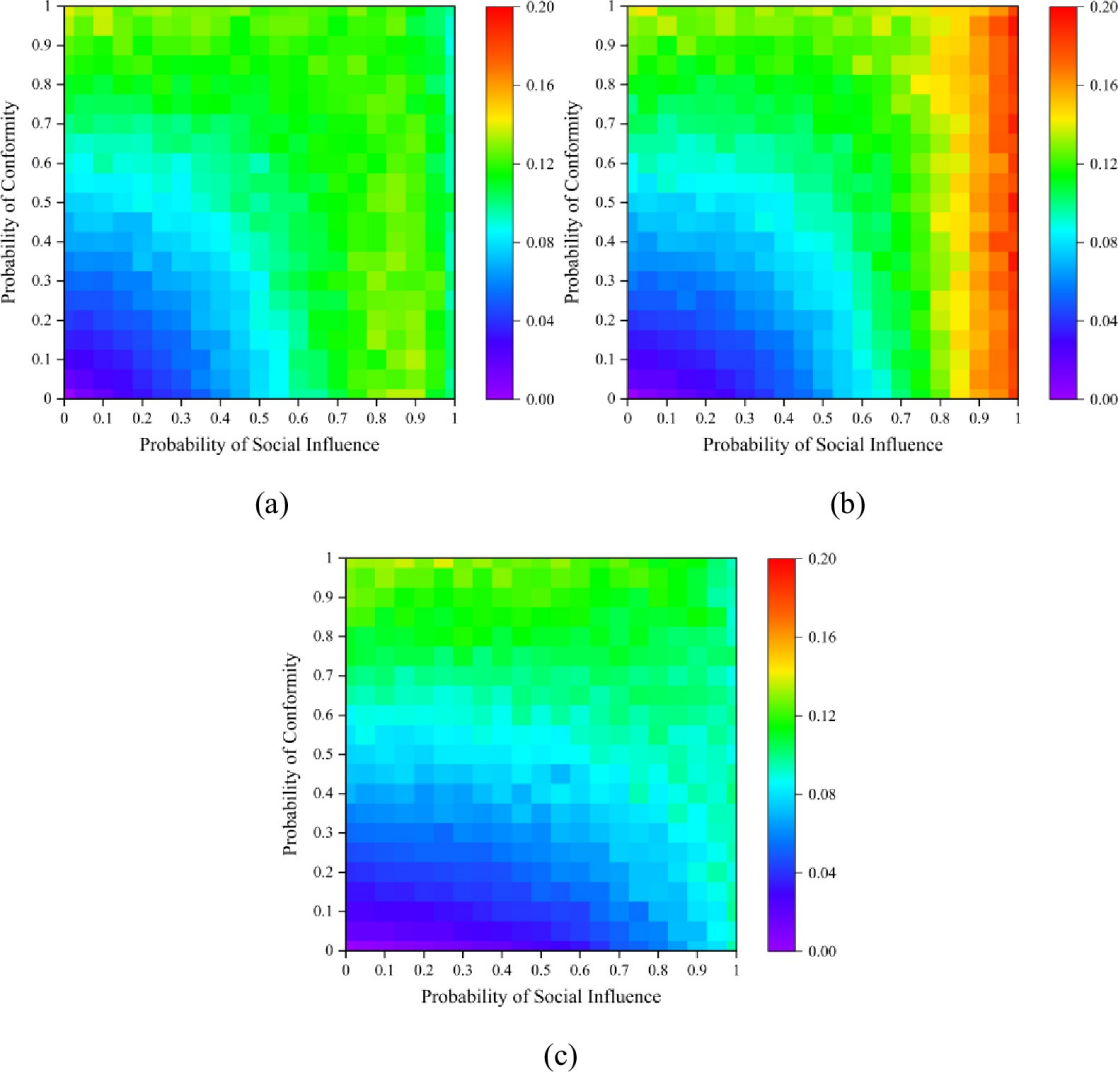
Unpredictability results under different probability of social influence *p*_*s*_ and probability of conformity *p*_*c*_. (a) is the result with a false start in a hypernetwork; (b) is an equal start in a hypernetwork; (c) is a correct start in a hypernetwork. We fix *δ*_1_ = 0.5.

Moreover, we explore the unpredictability of the dynamics. The measure of unpredictability *u*_*s*_ for each color *s* can be formulated as $u_{s}=\sum_{i=1}^{W}\sum_{j=i+1}^{W}\left|M_{s,i}-M_{s,j}\right|/(\begin{array}{c} W\\ 2 \end{array})$, where *M*_*s*,*i*_ represents the market share of color *s* in the *i*th experiment, and *W* = 500 represents the total number of independent world in the simulation process. The overall unpredictability is then computed as *U* = ∑*u*_*s*_/*S* across all colors.^16^ Lower unpredictability values indicate that the results tend to converge to a fixed point, namely, the evolution of the dynamic is less path-dependent. Fig 5 illustrates the results of the unpredictability analysis corresponding to Fig 4. In a hypernetwork structure, the unpredictability value increases with social influence *p*_*s*_ growing, and the equal start are more unpredictable than the correct and false start, confirming that neutrality leads to greater information entropy. In addition, when the social influence *p*_*s*_ and *p*_*c*_ are relatively small, the market is an independent world where all individuals will choose their preferred ball, the market will be completely predictable. As the social influence *p*_*s*_ increases, the market share of each color becomes more unpredictable, and a larger conformity probability *p*_*c*_ will promote unpredictability in the hypernetwork. This result suggests that a higher level of social influence generates a larger market instability, while the existence of group identity is more likely to exacerbate the unpredictability of the social system.

**Fig 6:**
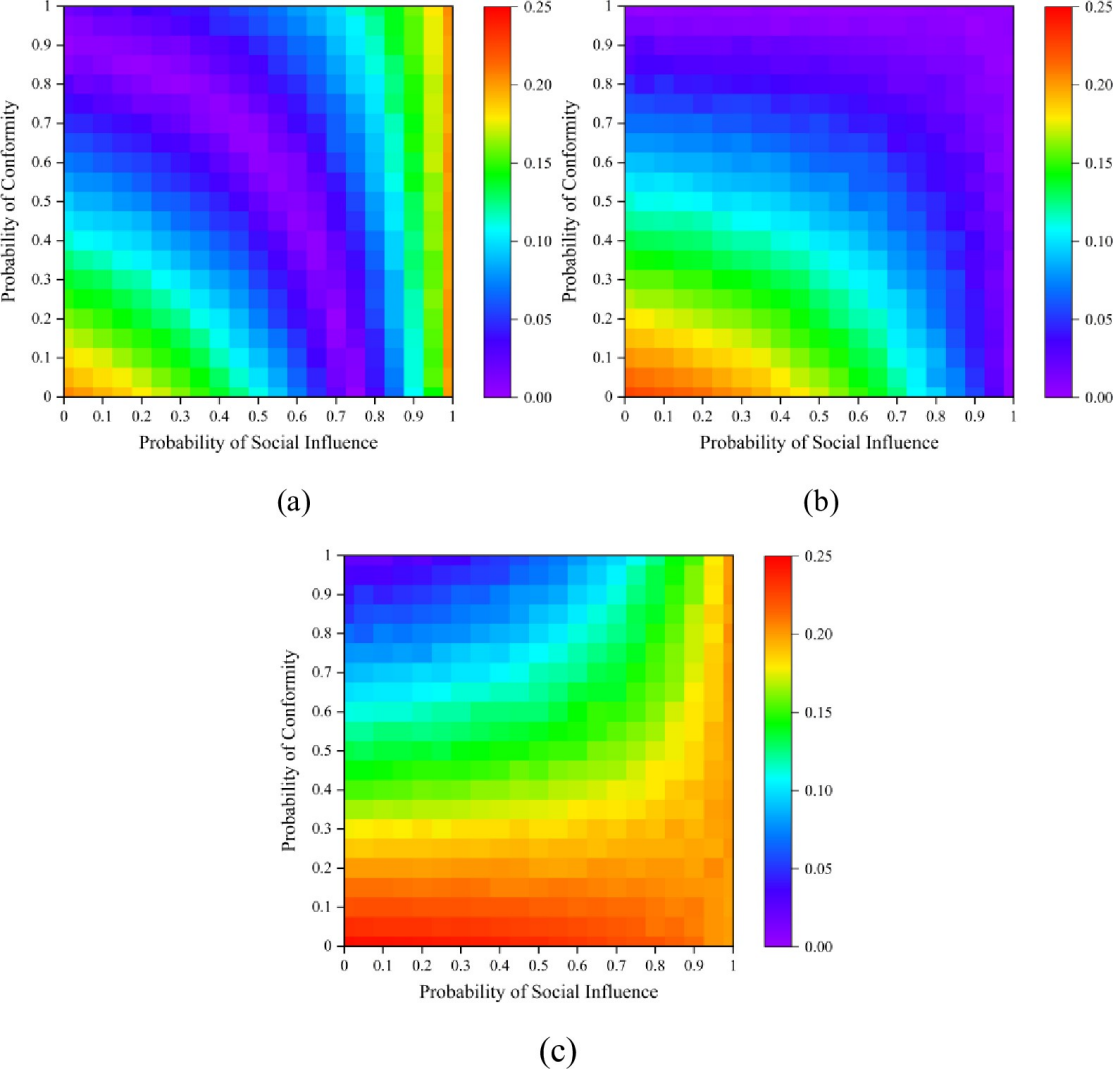
Inequality results for different probability of social influence *p*_*s*_ and probability of conformity *p*_*c*_. (a) is the result under a false start; (b) is the results under an equal start; (c) is the results under a correct start. We fix *δ*_1_ = 0.5.

We also investigate the inequality of the dynamics. The measure of inequality *G*_*s*_ for the social system is defined as $G_{s}=\sum_{i=1}^{N}\sum_{j=i+1}^{N}\left|M_{s,i}-M_{s,j}\right|/2N\sum_{k=1}^{N}M_{s,k}$. Parameter *N* indicates the number of choices available for individuals, here *N* equals to 2. The higher inequality between red balls and blue balls implies that the market is being disrupted by external effects, leading to what has been called “superstar” effect or “winner-take-all” markets [72, 73]. Fig 6 shows the results of the inequality analysis corresponding to Fig 4. Fig 6A illustrates that when the initial state of the market is a false state, the inequality results have a tendency to decrease then increase sequentially approximately along the diagonal. At the beginning, when both social influence *p*_*s*_ and the probability of conformity *p*_*c*_ are small, individual preference will let the red balls dominate in the market, leading to greater inequality. As the *p*_*c*_ value gradually increases, the effect of group identity will prompt a self-correcting process of the market, resulting in less inequality. However, when the probability of social influence *p*_*s*_ approaches 1, the false initial state allows blue balls to prevail, enlarging the value of market inequality again. The results in Fig 6B and 6C are quite different. When the initial state of the market is an equal start, higher inequality exists only when the values of both *p*_*s*_ and *p*_*c*_ are small, due to individual preference. While the initial state is a correct start, the market can self-correct to equality only when the *p*_*c*_ is large, otherwise the red balls will dominate the market.

The inequality results of *δ*_1_ = 0.3, 0.7 are shown in S1 and S2 Figs. Overall, the patterns of inequality results are similar. As *δ*_1_ becomes larger, the dominance of the red balls becomes more pronounced and the market tends to be more unequal.

**Fig 7:**
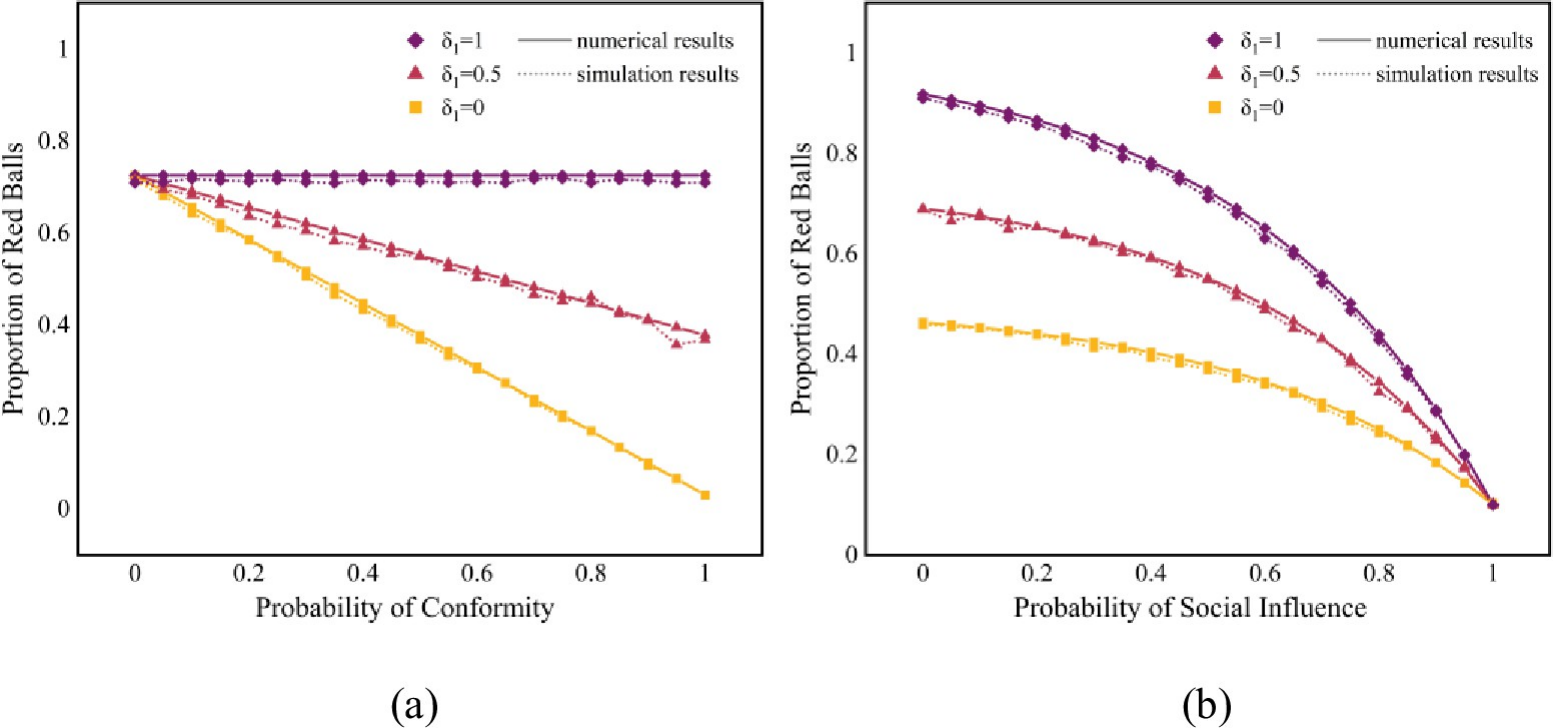
Comparison of numerical and simulation results. (a) are the results with fixed *p* = 0.5; (b) are the results with fixed *p*_*c*_ = 0.5. There are one red ball and nine blue balls at the initial.

In addition, we conduct several experiments focus on one certain hyperedge, which can be considered as a fully-connected network with group norms corresponding with the theoretical derivation. We set one red ball and nine blue balls as the initial state, with a total of 100 nodes. Fig 7 presents the comparison of numerical and simulation results with the change in *δ*_1_. The results for the initial states of equal start and correct start are presented S3 and S4 Figs, respectively. The simulation results are found to be in strict accordance with the numerical calculation results. The results in S5 Fig show that the dynamic results in the fully-connected state follow a similar pattern to those of the hypernetwork. However, the unpredictability results of the full-connected network shown in S6 Fig are significantly different from the unpredictability results of the hypernetwork. Holistically, when the system exhibits a higher extent of equality, the dynamic diffusion will be more divergent, manifesting in the fact that the unpredictability of the system will be greater when the initial state is an equal start or the initial purpose of the hyperedge is not explicit. Specifically, we found that fully-connected networks are far more unpredictable and this suggests different mechanisms of social influence diffusion in hypernetworks and fully-connected networks.

**Fig 8:**
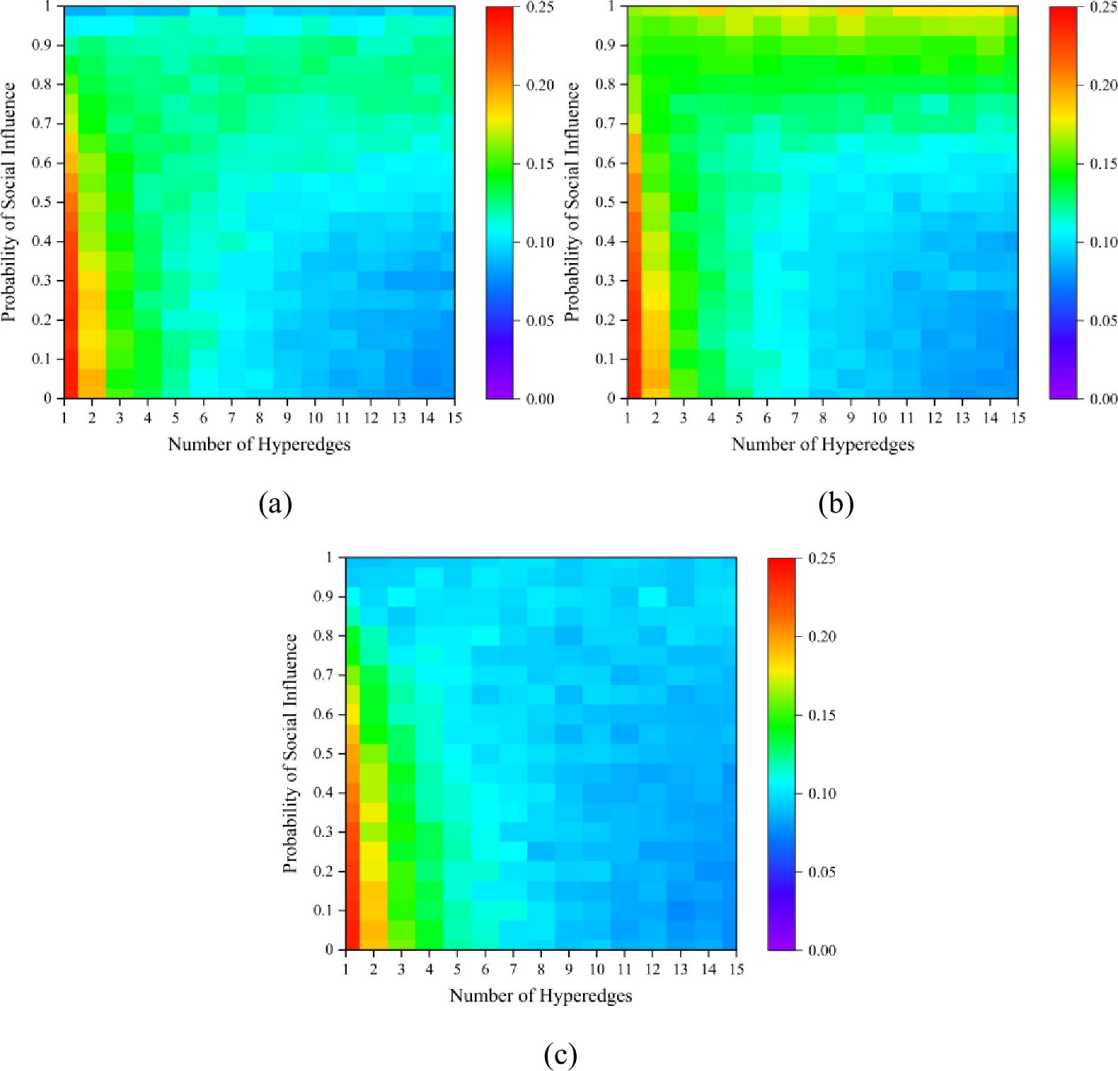
Unpredictability results for different numbers of hyperedges and probability of social influence *p*_*s*_. (a) is the result with a false start; (b) is an equal start; (c) is a correct start. We fix *δ*_1_ = 0.5, *p*_*c*_ = 0.5.

Ulteriorly, we explore the impact of the number of hyperedges on the dynamics. To ensure that the structure of hypernetworks with different numbers of hyperedges are comparable, we fix the number of nodes in this hypernetwork at 100 and ensure that they are all uniform hypernetworks. Each node has a 50% probability of belonging to two or more hyperedges; thus, for every newly generated hyperedge, it will randomly include some existing nodes. Simulation results with different numbers of hyperedges and probability of social influence are shown in S7 Fig. It can be seen that hypernetworks with different numbers of hyperedges exhibit a similar pattern of results. The dynamic results will come close to the initial state with larger social influence. Fig 8 shows that the unpredictability results of hypernetwork with different number of hyperedges are distinctly different. We fix *δ*_1_ = 0.5. Holistically, the hypernetwork structures with fewer hyperedges are more unpredictable. On the one hand, the effect of social influence in the hypernetworks with fewer hyperedges is much wider, which makes the dynamics more path-dependent regardless of the initial state. On the other hand, the hypernetworks with fewer hyperedges enable more paths for information diffusion, generating more uncertainty in regard to diffusion. Analogously, the identical conclusion can be drawn for the results of fixing the social influence probability while varying the value of conformity probability, and the results are shown in S8 and S9 Figs.

**Fig 9:**
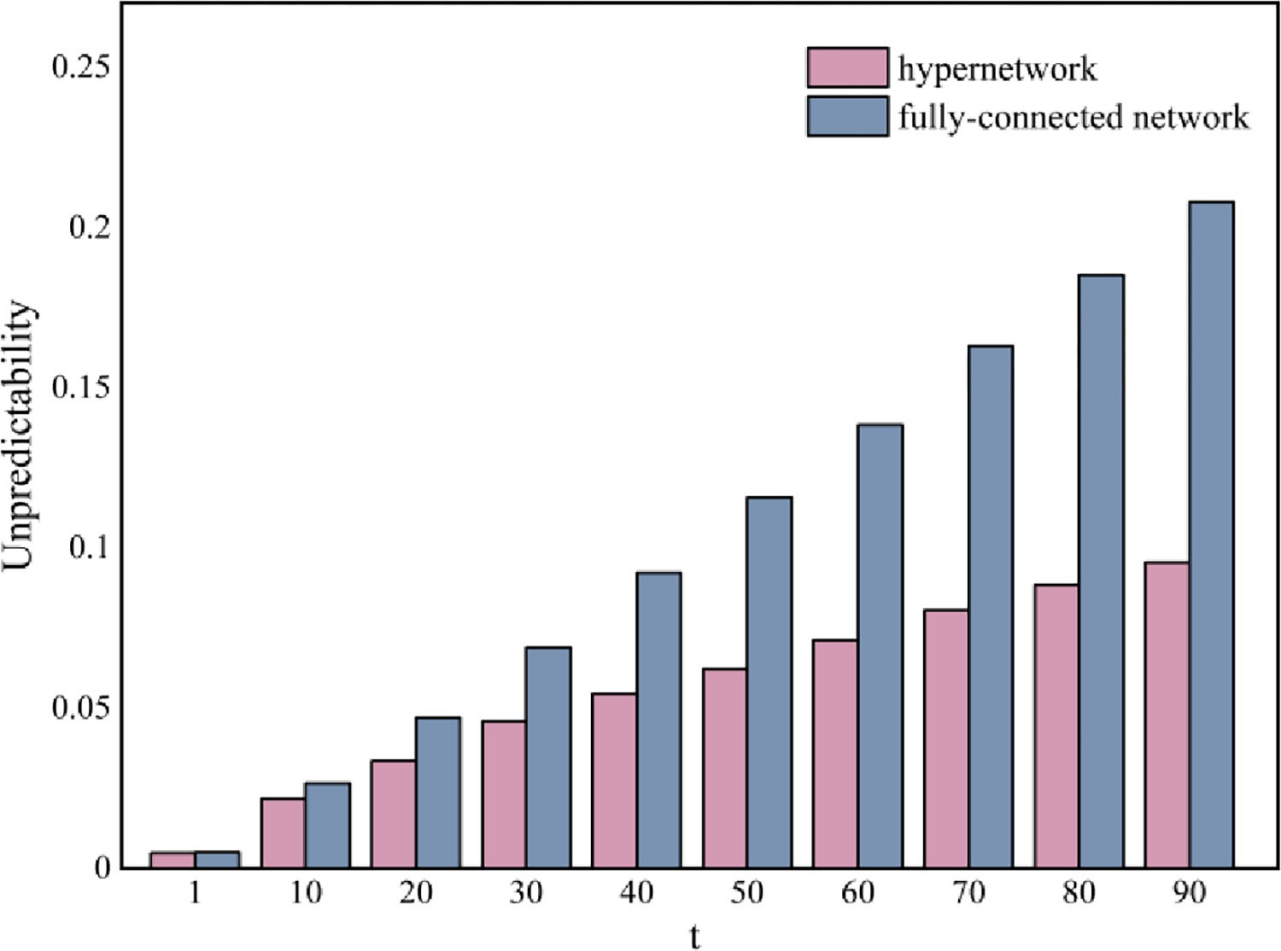
Unpredictability results at different diffusion times. Each diffusion time represents the moment when an individual makes a choice. We set a false start and fix *δ*_1_ = 0.5, *p*_*c*_ = 0.5, *p*_*s*_ = 0.5.

Particularly, we simulated the unpredictability value at different times in a fully-connected network and a hypernetwork as shown in Fig 9. The initialization settings are identical in both of two network structures. We can observe that the unpredictability of fully-connected networks is significantly higher than the unpredictability of hypernetworks, and becomes more pronounced over time. At the beginning, the low value of unpredictability is attributed to the low proportion of both red balls and blue balls, presenting a small scale of individual making decisions. The social system is relatively stable at this time. Subsequently, information is gradually spread across the entire system, the unpredictability value increases due to the multiplicity of diffusion paths. Fully-connected networks are much more unpredictable, and the way individuals receive information is more complex, which indicates the diffusion mechanisms of collective dynamics under different network structure are completely different. We can conclude that the diffusion paths of different network structures differ markedly, resulting in individuals behaving dissimilarly.

**Fig 10:**
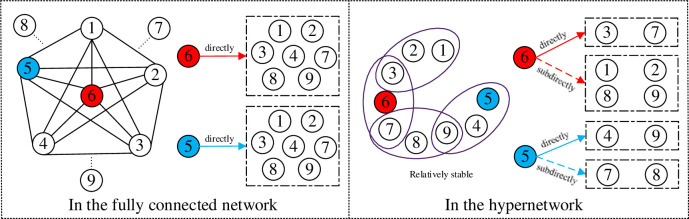
An illustration of diffusion paths in fully-connected networks and hypernetworks. In the left fully-connected network, individual 6 has numerous adjacent individuals, resulting in a more intricate diffusion path for its social influence compared to the hypernetwork.

A specific illustration of diffusion paths in a fully-connected network and a hypernetwork can be seen in Fig 10, which can explain why the unpredictability of the two network structures differs. If the diffusion path occurs in a fully-connected network, then at this moment, node 5 or node 6 directly affect seven nodes. In the next two diffusions, if the diffusion path follows the path 6→1→2, then node 1 and node 2 both chooses the red color; if the diffusion path follows the path 5→1→2, then node 1 and node 2 both chooses the blue color; if the diffusion path follows the path 6→1 5→2, then node 1 chooses the red color and node 2 chooses the blue color. As the number of nodes in the network increases, the choice of node 1 and node 2 will become far more complex and unpredictable. While if the diffusion process takes place in a hypernetwork, node 5 or node 6 only directly affect two nodes. As the number of hyperedges increases, the number of nodes in each hyperedge becomes smaller and the diffusion paths will be more predictable. Therefore, in the hypernetwork structure, the diffusion paths among nodes are relatively stable, resulting in lower unpredictability of the hypernetwork system.

## Conclusion and discussion

In this paper, we propose a hypernetwork-based urn model for explaining social influence, offering insights into the intricate dynamics of collective behavior. Our model also takes into account the impact of group identity, a critical factor in shaping the evolution of collective dynamics. The foundation of our investigation is rooted in the dynamic social impact theory introduced by Latané [74], which illuminates how individuals’ responsiveness to social influence increases in proportion to the influence’s source potency and cumulative number of sources. This theory serves as a robust underpinning for our exploration of collective dynamics. Complementing this, the conversion theory of social influence [75] aptly explains the group identity effect within social influence, highlighting the tendency of group members to converge. Furthermore, the theory of social structure [76] posits that social arrangements play a functional role within society, with each action serving a purpose toward desired outcomes. This theoretical framework lends substantial support to the feasibility of hypernetwork-structured social networks, wherein individuals operate within distinct organizational structures guided by a unified objective.

By integrating these theoretical constructs, we consider the individual preference and present a discussion of the crossover effect of group identity and social influence. We compute the unpredictability and inequality of collective dynamics under diverse settings. Additionally, we explore the principles of collective dynamics with different numbers of hyperedge, and interpret the differences of diffusion path between simple fully-connected network and hypernetwork structure.

Due to the effect of social influence, the choices of early decision-makers have a stronger impact on the latecomers, resulting in forming a self-reinforcement path in collective dynamics. Based on the simulation results, we confirm that social influence provides a strong imitation environment, which prevent the dynamics from being self-correcting. We conclude that the presence of social influence will make the market share converge to the level of the initial state, and social influence may render inferior-quality items more prevalent than superior items if biased perceptions of quality exist at the beginning. We also explore the effect of group identity, which enables individuals to perform in accordance with group norms. The predicted market share is highly congruent with group norms based on the mathematical deduction. The simulation results indicate that group identity has a moderating effect on the market, promoting dynamic equality. We also observe that the unpredictability value increases with the probability of social influence grows, and the equal start are more unpredictable than the correct state and false state, confirming that neutrality leads to greater information entropy. In addition, we conclude that the effect of group identity can moderate market inequality caused by individual preference and the effect of social influence. Group rationality would challenge individual values and drive collective dynamics in social systems toward non-sentimentalism. Moreover, we investigate that the patterns of collective dynamics result with different number of hyperedges are similar, but there are still differences in unpredictability results due to the complex diffusion paths under different network structures. The fully-connected networks are more unpredictable.

However, predicting market share remains difficult because of the uncertainties in the complex collective dynamics. Although our proposed models provide the theoretical and modeling basis for better understanding of the logic of collective dynamics, there are still several constraints in our models. In the future, we aim to explore the collective dynamics in the signed network by focusing on the specific mechanisms of homophily and xenophobia in the dynamics, and discuss the effect of different individual attributes on the dynamic results. Some real social system experiments should also be conducted hereafter to complement the practical connotations of social influence.

## Supporting information

S1 Fig Inequality results for different probability of social influence *p*_*s*_ and probability of conformity *p*_*c*_. (DOCX)

S2 Fig Inequality results for different probability of social influence *p*_*s*_ and probability of conformity *p*_*c*_. (DOCX)

S3 Fig Comparison of numerical and simulation results. (DOCX)

S4 Fig Comparison of numerical and simulation results. (DOCX)

S5 Fig The simulation results in a fully-connected network with different *δ*_1_. (DOCX)

S6 Fig Unpredictability results in the fully-connected network. (DOCX)

S7 Fig Proportion of the red balls with different numbers of hyperedges and probability of social influence *p*_*s*_. (DOCX)

S8 Fig Proportion of the red balls with different numbers of hyperedges and probability of conformity *p*_*c*_. (DOCX)

S9 Fig Unpredictability results for different numbers of hyperedges and probability of conformity *p*_*c*_. (DOCX)

## References

[1] Galesic M, Bruine de Bruin W, Dalege J, Feld SL, Kreuter F, Olsson H, et al. Human social sensing is an untapped resource for computational social science. Nature. 2021; 595: 214–222. doi: 10.1038/s41586-021-03649-2 34194037

[2] Sarkar K, Sundaram H. Influencing Busy People in a Social Network. PLoS ONE. 2016; 11: e0162014. doi: 10.1371/journal.pone.0162014 27711127 PMC5053606

[3] Dholakia UM, Bagozzi RP, Pearo LK. A social influence model of consumer participation in network-and small-group-based virtual communities. Int. J. Res. Mark. 2004; 21: 241–263. 10.1016/j.ijresmar.2003.12.004

[4] Chen W, Wang Y, Yang S. Efficient influence maximization in social networks. 15th ACM SIGKDD International Conference on Knowledge Discovery and Data Mining (KDD). 2009; pp. 199–208. 10.1145/1557019.1557047

[5] Ye M, Liu X, Lee WC. Exploring social influence for recommendation: a generative model approach. 35th International ACM SIGIR Conference on Research and Development in Information Retrieval (SIGIR). 2012; pp. 671–680. 10.1145/2348283.2348373

[6] Frey V, van de Rijt A. Social Influence Undermines the Wisdom of the Crowd in Sequential Decision Making. Manage. Sci. 2020; 67: 4273–4286. 10.1287/mnsc.2020.3713

[7] Li H, Meng F, Jeong M, Zhang Z. To follow others or be yourself? Social influence in online restaurant reviews. Int. J. Contemp. Hosp. M. 2020; 32: 1067–1087. 10.1108/IJCHM-03-2019-0263

[8] Popenoe D. Sociology: Study Guide, Prentice Hall. 1991.

[9] Macy MW, Willer R. From factors to actors: Computational sociology and agent-based modeling. Annu. Rev. Sociol. 2002; 28: 143–166. 10.1146/annurev.soc.28.110601.141117

[10] Sauermann J. Median voter dynamics in a laboratory experiment on voting over redistribution. Soc. Sci. Res. 2023; 111: 102869. doi: 10.1016/j.ssresearch.2023.102869 36898788

[11] Smith ER, Conrey FR. Agent-based modeling: A new approach for theory building in social psychology. Pers. Soc. Psychol. Rev. 2007; 11: 87–104. doi: 10.1177/1088868306294789 18453457

[12] Bou Zeineddine F, Leach CW. Feeling and thought in collective action on social issues: Toward a systems perspective. Soc. Personal. Psychol. Compass. 2021; 15: e12622. 10.1111/spc3.12622

[13] DeDeo S. Collective Phenomena and Non-Finite State Computation in a Human Social System. PLoS ONE. 2013; 8: e75818. doi: 10.1371/journal.pone.0075818 24130745 PMC3794014

[14] Sieben A, Schumann J, Seyfried A. Collective phenomena in crowds—Where pedestrian dynamics need social psychology. PLoS ONE. 2017; 12: e0177328. doi: 10.1371/journal.pone.0177328 28591142 PMC5462364

[15] Bak-Coleman JB, Alfano M, Barfuss W, Weber EU. Stewardship of global collective behavior. Proc. Natl. Acad. Sci. 2021; 118: e2025764118. doi: 10.1073/pnas.2025764118 34155097 PMC8271675

[16] Salganik MJ, Dodds PS, Watts DJ. Experimental Study of Inequality and Unpredictability in an Artificial Cultural Market. Science. 2006; 311: 854–856. doi: 10.1126/science.1121066 16469928

[17] Salganik MJ, Watts DJ. Leading the Herd Astray: An Experimental Study of Self-fulfilling Prophecies in an Artificial Cultural Market. Soc. Psychol. Q. 2008; 71: 338–355. 10.1177/019027250807100404 PMC3785310 24078078

[18] Sorensen AT. Bestseller lists and product variety. J. Ind. Econ. 2007; 55: 715–738. 10.1111/j.1467-6451.2007.00327.x

[19] Castellano C, Marsili M, Vespignani A. Nonequilibrium phase transition in a model for social influence. Phys. Rev. Lett. 2000; 85: 3536. doi: 10.1103/PhysRevLett.85.3536 11030940

[20] Sasahara K, Chen W, Peng H, Ciampaglia GL, Flammini A, Menczer F. Social influence and unfollowing accelerate the emergence of echo chambers. J. Comput. Soc. Sc. 2021; 4: 381–402. 10.1007/s42001-020-00084-7

[21] Arthur WB. Competing technologies, increasing returns, and lock-in by historical events. Econ. J. 1989; 99: 116–131. 10.2307/2234208

[22] Pemantle R. A survey of random processes with reinforcement. Probability surveys. 2007; 4: 1–79. doi: 10.1214/07-PS094

[23] Pólya G. Sur quelques points de la théorie des probabilités. Annales de l’institut Henri Poincaré. 1930; 1: 117–161. Availiable from: http://www.numdam.org/item?id=AIHP_1930__1_2_117_0

[24] Adler M. Stardom and Talent. Am. Econ. Rev. 1985; 75: 208–212. https://www.jstor.org/stable/1812714

[25] Hino M, Irie Y, Hisakado M, Takahashi T, Mori S. Detection of phase transition in generalized Polya urn in Information cascade experiment. J. Phys. Soc. Jpn. 2016; 85: 034002. 10.7566/JPSJ.85.034002

[26] He X, Lu J, Du H, Jin X. Social Influence in Signed Networks. IEEE Trans. Comput. Soc. Syst. 2023. doi: 10.1109/TCSS.2022.3220944

[27] Yadav AC. Critical Pólya urn. Phys. Rev. E. 2018; 98: 022119. 10.1103/PhysRevE.98.022119 30253581

[28] Hoppe FM. The sampling theory of neutral alleles and an urn model in population genetics. J. Math. Biol. 1987; 25: 123–159. doi: 10.1007/BF00276386 3611978

[29] Martin CF, Ho YC. Value of information in the Polya urn process. Inf. Sci. 2002; 147: 65–90. 10.1016/S0020-0255(02)00210-4

[30] Tria F, Loreto V, Servedio VDP, Strogatz H. The dynamics of correlated novelties. Sci. Rep. 2014; 4: 1–8. doi: 10.1038/srep05890 25080941 PMC5376195

[31] Lau PL, Koo TTR, Wu CL. Spatial distribution of tourism activities: A polya urn process model of rank-size distribution. J. Travel Res. 2020; 59: 231–246. 10.1177/0047287519829258

[32] Soto V, Suárez A, Martínez-Muñoz G. An urn model for majority voting in classification ensembles. Advances in Neural Information Processing Systems 29 (NIPS). 2016. Availibable from: https://proceedings.neurips.cc/paper_files/paper/2016

[33] Pastor-Satorras R, Vespignani A. Epidemic spreading in scale-free networks. Phys. Rev. Lett. 2001; 86: 3200–3203. doi: 10.1103/PhysRevLett.86.3200 11290142

[34] Gao L, Wang W, Pan L, Tang M, Zhang HF. Effective information spreading based on local information in correlated networks. Sci. Rep. 2016; 6: 38220. doi: 10.1038/srep38220 27910882 PMC5133588

[35] Liu C, Zhang ZK. Information spreading on dynamic social networks. Commun. Nonlinear Sci. Numer. Simul. 2014; 19: 896–904. 10.1016/j.cnsns.2013.08.028

[36] Estrada E, Rodríguez-Velázquez JA. Subgraph centrality and clustering in complex hyper-networks. Physica A. 2006; 364: 581–594. 10.1016/j.physa.2005.12.002

[37] Mao C, Xu C, He Q. A cost-effective algorithm for inferring the trust between two individuals in social networks. Knowledge-Based Syst. 2019; 164: 122–138. 10.1016/j.knosys.2018.10.027

[38] Friedkin NE. Norm formation in social influence networks. Soc. Networks. 2001; 23: 167–189. 10.1016/S0378-8733(01)00036-3

[39] Postmes T, Haslam SA, Swaab RI. Social influence in small groups: An interactive model of social identity formation. Eur. Rev. Soc. Psychol. 2005; 16: 1–42. 10.1080/10463280440000062

[40] Cinelli M, De Francisci Morales G, Galeazzi A, Starnini M. The echo chamber effect on social media. Proc. Natl. Acad. Sci. 2021; 118: e2023301118. doi: 10.1073/pnas.2023301118 33622786 PMC7936330

[41] Amini A, Firouzkouhi N, Gholami A, Gupta AR, Cheng C, Davvaz B. Soft hypergraph for modeling global interactions via social media networks. Expert. Syst. Appl. 2022; 203: 117466. 10.1016/j.eswa.2022.117466

[42] Gao J, Buldyrev SV, Stanley HE, Havlin S. Networks formed from interdependent networks. Nat. Phys. 2012; 8: 40–48. 10.1038/nphys2180 23005189

[43] Berge C. Graphsx and hypergraphs. North-Holland Publishing Co., Amsterdam. 1973.

[44] Ghoshal G, Zlatić V, Caldarelli G, Newman MEJ. Random hypergraphs and their applications. Phys. Rev. E. 2009; 79: 066118. doi: 10.1103/PhysRevE.79.066118 19658575

[45] Xiao Q. A method for measuring node importance in hypernetwork model. Res. J. Appl. Sci. 2013; 5: 568–573. doi: 10.19026/rjaset.5.4991

[46] Heintz B, Chandra A. Beyond graphs: toward scalable hypergraph analysis systems. ACM SIGMETRICS Performance Evaluation Review. 2014; 41: 94–97. 10.1145/2627534.2627563

[47] Bodó Á, Katona GY, Simon PL. SIS epidemic propagation on hypergraphs. Bull. Math. Biol. 2016; 78: 713–735. doi: 10.1007/s11538-016-0158-0 27033348

[48] Burgio G, Matamalas GT, Gómez S, Arenas A. Evolution of cooperation in the presence of higher-order interactions: from networks to hypergraphs. Entropy. 2020; 22: 744. doi: 10.3390/e22070744 33286516 PMC7517288

[49] Golubski AJ, Westlund EE, Vandermeer J, Pascual M. Ecological networks over the edge: hypergraph trait-mediated indirect interaction (TMII) structure. Trends. Ecol. Evol. 2016; 31: 344–354. doi: 10.1016/j.tree.2016.02.006 26924738

[50] Ma YJ, Ma YY. Hypergraph-based logistic matrix factorization for metabolite–disease interaction prediction. Bioinformatics. 2022; 38: 435–443. doi: 10.1093/bioinformatics/btab652 34499104

[51] La Gatta V, Moscato V, Pennone M, Postiglione M, Sperlí G. Music Recommendation via Hypergraph Embedding. IEEE Trans. Neural Netw. Learn Syst. 2022. doi: 10.1109/TNNLS.2022.3146968 35143406

[52] Civilini A, Anbarci N, Latora V. Evolutionary game model of group choice dilemmas on hypergraphs. Phys. Rev. Lett. 2021; 127: 268301. doi: 10.1103/PhysRevLett.127.268301 35029481

[53] Wang JW, Rong LL, Deng QH, Zhang JY. Evolving hypernetwork model. Eur. Phys. J. B. 2010; 77: 493–498. 10.1140/epjb/e2010-00297-8

[54] Hu F, Zhao HX, Ma XJ. An evolving hypernetwork model and its properties. Sci. Sin. Phys. Mech. Astron. 2013; 43: 16–22. doi: 10.1360/132012-87

[55] Johnson J. Hypernetworks in the science of complex system*s*. Imperial College Press. 2014. doi: 10.1142/9781860949739_0006

[56] Chen MR, Kuba M. On generalized Pólya urn models. J. Appl. Probab. 2013; 50: 1169–1186. 10.1239/jap/1389370106

[57] Gouet R. A martingale approach to strong convergence in a generalized Pólya-Eggenberger urn model. Stat. Probabil. Lett. 1989; 8: 225–228. 10.1016/0167-7152(89)90126-0

[58] Schreiber SJ. Urn models, replicator processes, and random genetic drift. SIAM J. Appl. Math. 2001; 61: 2148–2167. 10.1137/S0036139999352857

[59] González-Navarrete M, Lambert R. Urn models with two types of strategies. arXiv preprint arXiv:1708.06430. 2017. 10.48550/arXiv.1708.06430

[60] Noonan J, Lambiotte R. Dynamics of majority rule on hypergraphs. Phys. Rev. E. 2021; 104: 024316. doi: 10.1103/PhysRevE.104.024316 34525590

[61] Olson M. The logic of collective action. Harvard University Press. 1965.

[62] Spears R. Social influence and group identity. Annu. Rev. Psychol. 2021; 72: 367–390. doi: 10.1146/annurev-psych-070620-111818 32931718

[63] McClain PD, Johnson Carew JD, Walton E Jr., Watts CS. Group membership, group identity, and group consciousness: Measures of racial identity in American politics. Annu. Rev. Polit. Sci. 2009; 12: 471–485. 10.1146/annurev.polisci.10.072805.102452

[64] Gurin P, Miller AH, Gurin G. Stratum identification and consciousness. Soc. Psychol. Q. 1980; 43: 30–47. 10.2307/3033746

[65] Conover PJ. The role of social groups in political thinking. Br. J. Polit. Sci. 1988; 18: 51–76. 10.1017/S0007123400004956

[66] Chen Y, Li SX. Group identity and social preferences. Am. Econ. Rev. 2009; 99: 431–457. doi: 10.1257/aer.99.1.431

[67] Robins G, Pattison P, Elliott P. Network models for social influence processes. Psychometrika. 2011; 66: 161–189. 10.1007/BF02294834

[68] McPherson JM. Hypernetwork sampling: Duality and differentiation among voluntary organizations. Soc. Networks. 1982; 3: 225–249. 10.1016/0378-8733(82)90001-6

[69] Chan WKV, Hsu C. Service value networks: humans hypernetwork to cocreate value. IEEE T. Syst. Man Cy. A. 2012; 42: 802–813. doi: 10.1109/TSMCA.2012.2183356

[70] Albert S, Whetten DA. Organizational identity. From Research in Organizational Behavior. 1985; 7: 263–295.

[71] Suo Q, Guo JL, Shen AZ. Information spreading dynamics in hypernetworks. Physica A. 2018; 495: 475–487. 10.1016/j.physa.2017.12.108

[72] Rosen S. The economics of superstars. Am. Econ. Rev. 1981; 71: 845–858. Available from: https://www.jstor.org/stable/1803469

[73] Frank RH, Cook PJ. The winner-take-all society: Why the few at the top get so much more than the rest of us. Random House. 2010.

[74] Latané B. Dynamic social impact: The creation of culture by communication. J. Commun. 1996; 46(4): 13–25. 10.1111/j.1460-2466.1996.tb01501.x

[75] Moscovici S. Toward a theory of conversion behavior. Adv. Exp. Soc. Psychol. 1980; 13: 209–239. 10.1016/S0065-2601(08)60133-1

[76] Merton R K. Social theory and social structure. The Free Press. 1968.

