# Supplementary material for: A hypernetwork-based urn model for explaining collective dynamics
Source: PLoS One. 2023 Sep 19;18(9):e0291778. doi: 10.1371/journal.pone.0291778 (PMC10508602; doi:10.1371/journal.pone.0291778)
Supplement: S3 Fig — (DOCX) [file pone.0291778.s003.docx]

(a) (b)

S3 Fig. Comparison of numerical and simulation results. (a) are the results with fixed $p=0.5$; (b) are the results with fixed$p_{c}=0.5$. There are five red balls and five blue balls at the initial.
