# Supplementary material for: A hypernetwork-based urn model for explaining collective dynamics
Source: PLoS One. 2023 Sep 19;18(9):e0291778. doi: 10.1371/journal.pone.0291778 (PMC10508602; doi:10.1371/journal.pone.0291778)
Supplement: S5 Fig — (DOCX) [file pone.0291778.s005.docx]

(a) (b)





(c)

S5 Fig. The simulation results in a fully-connected network with different$\delta_{1}$. (a) is the simulation result with $\delta_{1}=0$; (b) is the simulation result with $\delta_{1}=0.5$; (c) is the simulation result with$\delta_{1}=1$. There are one red ball and nine blue balls at the initial.
