# Supplementary material for: A hypernetwork-based urn model for explaining collective dynamics
Source: PLoS One. 2023 Sep 19;18(9):e0291778. doi: 10.1371/journal.pone.0291778 (PMC10508602; doi:10.1371/journal.pone.0291778)
Supplement: S7 Fig — (DOCX) [file pone.0291778.s007.docx]

(a) (b)





(c)

S7 Fig. Proportion of the red balls with different numbers of hyperedges and probability of social influence *p_s_*. (a) is the result with a false start; (b) is an equal start; (c) is a correct start. We fix $\delta_{1}=0.5$, $p_{c}=0.5$.
