# Supplementary material for: A hypernetwork-based urn model for explaining collective dynamics
Source: PLoS One. 2023 Sep 19;18(9):e0291778. doi: 10.1371/journal.pone.0291778 (PMC10508602; doi:10.1371/journal.pone.0291778)
Supplement: S9 Fig — (DOCX) [file pone.0291778.s009.docx]

(a) (b)





(c)

S9 Fig. Unpredictability results for different numbers of hyperedges and probability of conformity *p_c_*. (a) is the result with a false start; (b) is an equal start; (c) is a correct start. We fix $\delta_{1}=0.5$, $p=0.5$.
